# Supplementary material for: An Embedded Mixed-Methods Study with a Dominant Quantitative Strand: The Knowledge of Jordanian Mothers About Risk Factors for Childhood Hearing Loss
Source: Audiol Res. 2025 Jul 16;15(4):87. doi: 10.3390/audiolres15040087 (PMC12286161; doi:10.3390/audiolres15040087)
Supplement: Supplementary file 1 [file audiolres-15-00087-s001.zip › audiolres-3638378-supplementary.pdf]

Questionnaire for Maternal Knowledge of Hearing Loss Risk Factors

Section 1: Control Variables

1. **What is your (mothers') highest level of education?**
   - No formal education
   - Primary school
   - Secondary school
   - College diploma
   - University degree or higher
2. **What is your household's monthly income level?** *(Socioeconomic Status - SES)*
   - Low (below average)
   - Moderate (average)
   - High (above average)

Section 2: Maternal Knowledge of Risk Factors for Hearing Loss (Independent Variable)

Instructions: Please indicate how much you agree with the following statements. *(1 = Strongly Disagree, 5 = Strongly Agree)*

1. Hearing loss can occur in children from infections of pregnancy (e.g., rubella, cytomegalovirus).
2. Consanguineous (close relative) marriage is a risk factor for hearing loss in children.
3. Children can have their hearing loss caused by exposure to high levels of noise.
4. Hearing problems in babies can occur as a result of ototoxic (harmful to the ear) medications during pregnancy.
5. Hearing loss is more likely to occur when a baby is born prematurely or is born at a low birth weight.
6. A high maternal fever during pregnancy can lead to a baby with hearing problems.
7. Infants with a family history of congenital hearing loss are the strongest predictor of hearing loss.

Section 3: Accuracy & Comprehensiveness of Maternal Knowledge (Dependent Variable)

Instructions: Please indicate your level of confidence in understanding the following hearing loss risk factors.

1. I am quite sure of identifying infections that lead to pregnancy hearing loss infections.
2. I know how marriage among consanguineous may raise the risk of hearing impairment.
3. I am aware that loud noises can damage a child's hearing.
4. I know how ototoxic medications can affect a baby's hearing development.
5. I am aware of the symptoms of early hearing loss in my child.
6. I understand what medical conditions (such as the jaundice, premature birth) can affect the development of hearing.
7. I am aware of the healthcare services for diagnosis and treatment of childhood hearing loss.

Section 4: Health Literacy Level (Moderating Variable)

Instructions: Please indicate how often the following statements apply to you.

1. I easily understand health related information given by doctors and nurses.
2. I am confident about asking healthcare professionals anything regarding my child's health.
3. I can read and understand medical instructions such as prescriptions and health brochures.
4. I can observe medical advice given by healthcare providers related to the hearing of my child.
5. I know how to make decisions about things in my health, this is all based on what I know.
6. I can easily explain some of the health related topics to my relatives.
7. I will ask for clarifications on medical information if I do not understand it.

Interview Questions

1. What would you say are the main reasons that hearing loss occurs in children?
2. Have you ever heard about the risk factors for the hearing loss from doctors, media or any other family? If this is the case, how trustworthy do you think that information is?
3. How sure are you in detecting early signs of hearing loss in a child?
4. What challenges do you experience trying to understand a patient's medical advice concerning hearing loss, or child health?
5. What role does your education play in how you comprehend the health risks, including hearing loss?
6. Does financial status matter when it comes to mothers' access to information, or treatment for, childhood hearing loss? If so, how?
7. How would you go about if your child were to be diagnosed with a hearing problem?
8. What could be done to improve mothers' awareness of hearing loss risk factors?
